# Survey of acute exacerbation after nonpulmonary surgery in patients with interstitial pneumonia

**DOI:** 10.1186/s40981-021-00433-z

**Published:** 2021-04-01

**Authors:** Miho Hamada, Ryuichi Wakata, Misaki Saito Sato, Toshiyuki Mizota

**Affiliations:** Department of Anesthesia, Kyoto University Hospital, 54 Shogoin-Kawahara-Cho, Sakyo-Ku, Kyoto, 606-8507 Japan

**To the Editor:**

Acute exacerbation (AE) of interstitial pneumonia (IP) is described as an acute respiratory deterioration accompanied by newly developed bilateral ground-glass opacity and/or consolidation identified on chest radiographs or computed tomography (CT) scans. It occurs in 9.3–15.8% of patients with IP undergoing pulmonary surgery and has a high fatality rate [1–3]. In contrast, limited data exists on the incidence of AE after nonpulmonary surgery; only three reports have been published so far, and the reported incidence deviates from 1.4 to 6.3% [4–6]. One of these reports compared the incidences of AE after pulmonary and nonpulmonary surgeries but noted no significant difference [4]. In this study, we examined the incidence of AE within 30 days after nonpulmonary surgery in patients with IP.

Figure 1 shows the flow diagram of this study. We included 220 patients with IP undergoing nonpulmonary surgery under general anesthesia at Kyoto University Hospital between 2008 and 2017. Patients with IP were identified by screening using the 10th revision of the International Statistical Classification of Diseases and Related Health Problems (ICD-10) diagnosis codes, followed by a review of chest CT reports. The diagnosis of AE was made based on the same criteria used in previous studies: (1) new bilateral ground-glass opacity/consolidation identified on chest CT, (2) acute worsening or development of dyspnea noted in the medical records, and (3) exclusion of heart failure, excessive fluid infusion, or lung infection as causes [4–6].

**Fig. 1:**
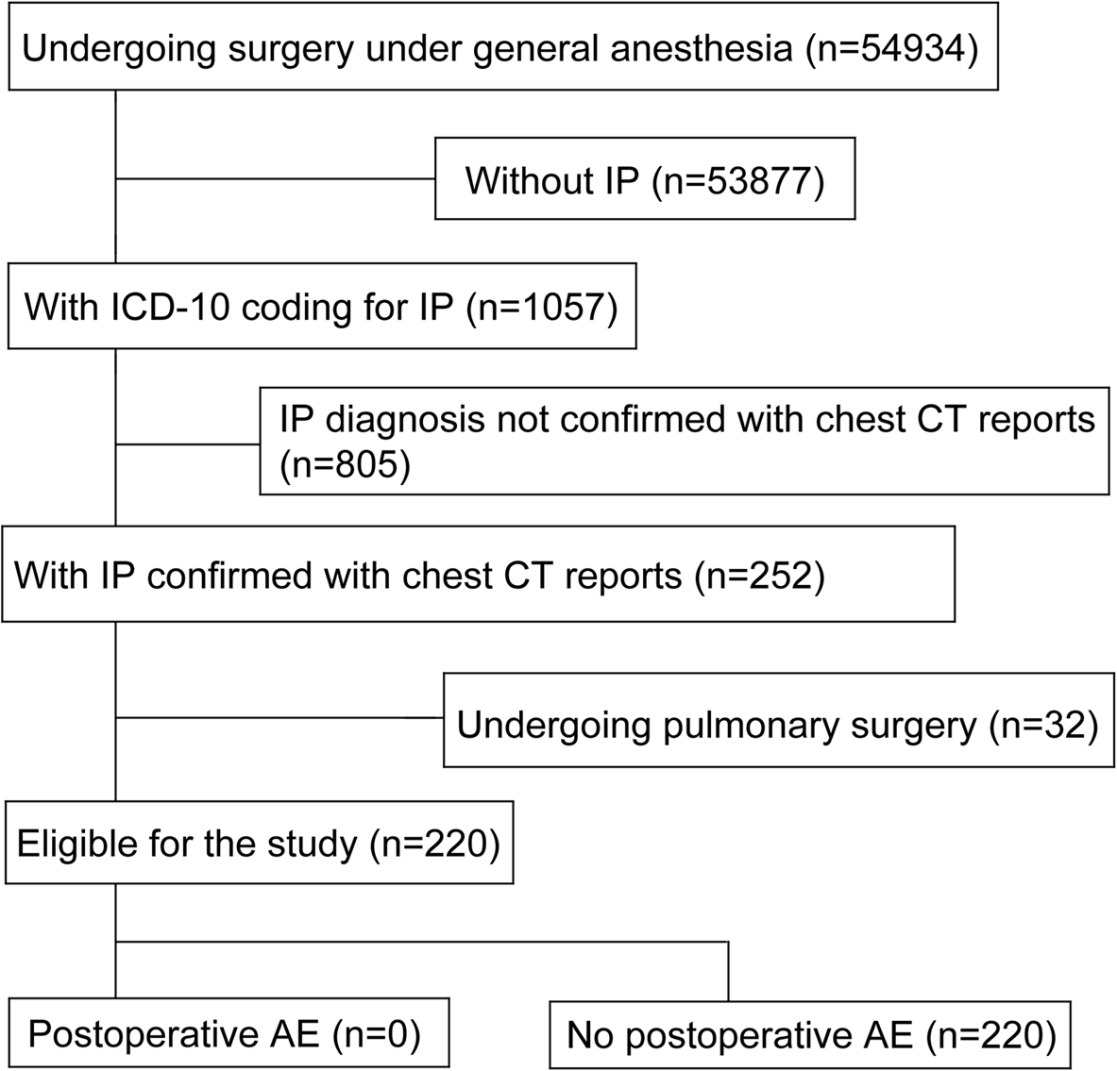
Flow diagram of the study participants. IP interstitial pneumonia, ICD International Classification of Disease, CT computed tomography, AE acute exacerbation

Table 1 shows the characteristics and operative variables of patients included in this study. No patients who underwent nonpulmonary surgery reported AE within 30 days after surgery; two patients presented with new infiltrative shadows on chest CT within 30 days after surgery, both of which were unilateral and caused by lung infection. The incidence of AE after nonpulmonary surgery was estimated to be 1.5% (95% confidence interval: 0.9–2.4%), when the result of this study was integrated with those of previous studies [4–6]. On the other hand, one of the 32 patients with IP undergoing pulmonary surgery had AE within 30 days after surgery.

**Table 1:** Characteristics and operative variables of patients included in this study who underwent nonpulmonary and pulmonary surgeries

Characteristics	Nonpulmonary surgeries (*n* = 220)	Pulmonary surgeries (*n* = 32)
Age (years)	70 [62–77]	61 [43–74.5]
Males	114 (52%)	26 (81.3%)
Body mass index (kg/m^2^)	21.7 [19.0–23.9]	22.3 [18.2–24.1]
Ever smoker	74 (33.6%)	16 (50%)
Past acute exacerbation	1 (0.5%)	0 (0%)
KL-6 (U/mL)	481 [319–778]	519 [336–789.5]
LDH (U/L)	212 [180–251]	211 [184.5–231.5]
CRP (mg/dL)	0.3 [0.1–1.6]	0.2 [0.1–0.7]
Usual interstitial pneumonia pattern	26 (11.8%)	2 (6.3%)
%VC (%)	84.5 [71.4–95.6]	96.5 [89.2–99.0]
FEV1/FVC (%)	94.8 [91.9–101.5]	96.1 [93.9–100.2]
%DLCO (%)	62.0 [44.1–81.2]	67.3 [62.5–78.5]
ASA-PS (1/2/3/4/missing)	6/119/60/9/23	2/15/10/4/1
Emergency surgery	31 (14.1%)	15 (46.9%)
Duration of anesthesia (min)	209.5 [139.5–349.0]	266 [167.5–381]
Blood loss (mL)	50 [0–214]	19 [0–430]

Continuous variables were presented as medians [interquartile range]. Categorical variables were presented as numbers (percentage). *KL-6* Klebs von den Lungen-6, *LDH* lactate dehydrogenase, *CRP* C-reactive protein, *VC* vital capacity, *FEV1/FVC* forced expiratory volume in 1 s/forced vital capacity, *DLCO* diffusing capacity for carbon monoxide, *ASA-PS* American Society of Anesthesiologists Physical Status Classification

The incidence of AE after nonpulmonary surgery estimated in this study tended to be lower than that reported in previous studies, possibly because fewer patients had known risk factors for postoperative AE, such as usual IP pattern, AE history, and a high C-reactive protein level (Table 1) [5]. The data of this study could reinforce the hypothesis that postoperative AE is less common in patients undergoing nonpulmonary surgery than those undergoing pulmonary surgery, although the previous study did not find any significant difference in the incidence of postoperative AE between pulmonary and nonpulmonary surgeries [4].

Due to various limitations, further validation of our findings is warranted. For example, few patients in this study had known AE risk factors, which may be due to the exclusion of high-risk patients from surgical treatment. Our study and all other previous studies have been carried out in Japan, thereby limiting the generalization of results to other settings.

## Data Availability

The datasets used and analyzed in this study are available from the corresponding author on a reasonable request.

## Abbreviations

AE: Acute exacerbation
IP: Interstitial pneumonia
CT: Computed tomography
ICD-10: International Statistical Classification of Diseases and Related Health Problems - 10th revision
